# Correlates of Physical Activity among Adults with Sight Loss in High-Income-Countries: A Systematic Review

**DOI:** 10.3390/ijerph182211763

**Published:** 2021-11-09

**Authors:** Rosie K. Lindsay, Francesco Di Gennaro, Peter M. Allen, Mark A. Tully, Claudia Marotta, Damiano Pizzol, Trish Gorely, Yvonne Barnett, Lee Smith

**Affiliations:** 1 Vision and Hearing Sciences Research Centre, Anglia Ruskin University, Cambridge CB1 1PT, UK; rkl109@pgr.aru.ac.uk (R.K.L.); peter.allen@aru.ac.uk (P.M.A.); 2 Department of Biomedical Sciences and Human Oncology, Clinic of Infectious Diseases, University of Bari “Aldo Moro”, 70124 Bari, Italy; cicciodigennaro@yahoo.it; 3 Institute of Mental Health Sciences, School of Health Sciences, Ulster University, Newtownabbey BT37 0QB, UK; m.tully@ulster.ac.uk; 4 General Directorate of Health Prevention, Ministry of Health, 00144 Rome, Italy; marotta.claudia@gmail.com; 5 Clinical Board, Cigna Health Insurance, 00185 Rome, Italy; 6 Centre for Health Science, Department of Nursing and Midwifery, University of the Highlands and Islands, Inverness IV2 3JH, UK; trish.gorely@uhi.ac.uk; 7 School of Life Sciences, Faculty of Science and Engineering, Anglia Ruskin University, Cambridge CB1 1PT, UK; yvonne.barnett@aru.ac.uk; 8 The Cambridge Centre for Sport and Exercise Science, Anglia Ruskin University, Cambridge CB1 1PT, UK; lee.smith@aru.ac.uk

**Keywords:** vision loss, visual impairment, physical activity, modifiable, non-modifiable, correlates

## Abstract

Background: Physical activity (PA) is essential for almost all facets of health; however, research suggests that PA levels among populations with sight loss are critically low. The aim of this review was to identify the correlates of PA among people with sight loss in high income countries, to inform future interventions and policies. Methods: MEDLINE, Web of Science, PsycINFO, SPORTDiscus, The British Journal of Visual Impairment, The Journal of Visual Impairment and grey literature were searched for studies which reported correlates of PA among adults with sight loss. The protocol is available from PROSPERO (CRD42020215596). Results: A total of 29 articles were eligible for review. Evidence from multiple studies reported that the vision impairment category, worse visual acuity, bilateral visual field loss, worse contrast sensitivity, those of the female gender, low self-efficacy, and environmental barriers were associated with lower levels of PA among populations with sight loss. Conclusions: Overall, correlates of PA among people with sight loss in high income countries are complex and vary across different population groups. Health professionals, eye care, and sight loss services should work together to identify people at risk of low PA, and provide a range of services and interventions to influence the modifiable factors that are associated with low PA.

## 1. Introduction

Physical activity (PA) is defined as ‘any movement produced by skeletal muscles that requires energy expenditure–including activities undertaken while working, playing, carrying out household chores, travelling, and engaging in recreational pursuits’ [1]. The World Health Organisation (WHO) recommends that adults should engage in at least 150 min of moderate PA or 75 min of vigorous PA, or an equivalent combination of both intensities throughout the week, these recommendations are the same for adults with disabilities [2]. Moderate PA can be defined as PA performed at 3–6 times the intensity of rest, whilst vigorous PA can be defined as PA performed at >6 times the intensity of rest and moderate–vigorous PA (MVPA) is PA performed at >3 the intensity of rest [2]. Regular and sustained participation in MVPA is beneficial for almost every aspect of physical and mental health. Indeed, there is a large body of literature that shows that regular and sustained participation in MVPA can aid in the prevention of a plethora of chronic conditions [3].

At least 2.2 billion people worldwide have a near or distance visual impairment (VI) [4]. Visual impairment is classified into distance and near presenting vision impairment. A distance vision impairment ranges from mild (visual acuity worse than 6/12 to 6/18) to blindness (visual acuity worse than 3/60). A near vision impairment is defined as near visual acuity worse than N6 or M.08 at 40cm [5]. In the UK, it is estimated that there are over 2 million people living with sight loss; this includes people with sight loss severe enough to be certified as visually impaired (sight impaired) or blind (severely sight impaired), as well as those with less severe sight loss. By 2050 the number of people living with sight loss in the UK is predicted to increase to over 4 million [6]. However, studies conducted in high income countries (HIC) have consistently reported that low levels of PA are associated with poorer vision [7,8]. For example, in a sample of older English adults, those who rated their eye-sight ‘fair–poor’ whilst wearing glasses or contact lenses were more than twice as likely to report being physically inactive than adults who reported having ‘excellent vision’ [9]. Low levels of PA are a concern as research has found that low levels of PA are associated with an increased risk of mortality in populations with sight loss [10,11,12].

Despite a global and growing population of people with sight loss, as well as the risks of low levels of PA, few studies have examined interventions aiming to promote PA among populations with sight loss. A systematic review of PA interventions for adults with VI found evidence that interventions can be beneficial, particularly for measures of balance and mobility [13]. However, in a subsequent meta-analysis that combined the results of four studies, the effect of PA interventions on mobility outcome measures were non-significant [13]. To inform the development of future interventions, it is important to first identify correlates of PA in those with sight loss.

Correlates can be either modifiable or non-modifiable. Modifiable correlates provide mechanisms to target for change (e.g., self-efficacy, accessible facilities), while non-modifiable correlates determine the target population for a PA intervention (e.g., visually impaired versus blind, additional impairments versus no additional impairment, males versus females or young versus old). People with sight loss represent a diverse population, and people’s experience of sight loss may depend on the severity of their sight loss, or the part of their sight which is impaired. For example, age-related macular degeneration predominantly affects central vision [14], whilst retinitis pigmentosa affects peripheral vision [15]; someone with either condition may have sight loss severe enough to be registered as VI, however both conditions affect sight differently. In addition, sight loss can occur across the sociodemographic spectrum, and sociodemographic factors and sight loss could intersect to influence PA either positively or negatively. 

However, to our knowledge no attempt has been made to understand the complexities of the factors which could be associated with PA among people with sight loss, which is needed to determine targeted interventions. Therefore, the aim of this review was to identify modifiable and non-modifiable correlates of PA among people with sight loss in high income countries (HICs). 

## 2. Materials and Methods

### 2.1. Search Strategy

The review followed the reporting guidelines outlined by the PRISMA checklist [16]. The protocol for the present review has been registered in the PROSPERO database (CRD42020215596). To identify studies which reported correlates with PA among populations with sight loss, electronic databases, reference lists, and grey literature were searched. We searched MEDLINE, Web of Science, PsycINFO, and SPORTDiscus for English language articles published from database inception to 1 October 2020. A subject librarian was consulted, and the search terms included terms related to physical activity AND correlates AND low vision (see Appendix A). The title and abstracts of all the search results were independently screened by two trained reviewers (RL and LS) for articles eligible for full text screening. The reference list of studies included in full text screening were also examined for eligible studies. The grey literature was searched from inception to November 2020 using the databases EThOS and Open Grey. The websites for the Royal National Institute of Blind People (RNIB), British Blind Sport, The Macular Society, Blind Veterans UK, Sight Savers, Guide Dogs for the Blind Association, Fight for Sight, SeeAbility, International Glaucoma Association (IGA), and World Sight Foundation were also searched for eligible studies. The British Journal of Visual Impairment, and The Journal of Visual Impairment and Blindness were also searched for the terms ‘exercise’, ‘physical activity’, and ‘sport’ in articles published from 2020–3 November 2021. 

### 2.2. Study Selection

Studies were included if they were cross-sectional or longitudinal observational studies that met the following inclusion criteria: (1) included adults aged 18 and over, or the study performed a sub-group analysis of adults (age 18 and over). (2) Studies were conducted among participants with sight loss whilst wearing corrective devices, either measured by clinical assessment or self-reported sight loss. (3) Studies conducted in HICs (defined as countries identified by the World Bank as high income in 2020). (4) The dependent variable was a measure of PA engagement (e.g., how often and for how long a person goes to a sports club or walks for transport). (5) The significance level of the association between PA and an independent variable was examined, and a *p*-value was reported. The review excluded studies conducted among populations under the age of 18, as correlates of PA among people under the age of 18 with sight loss is the focus of another review [17]. In addition, the review focused on correlates among HICs because these countries were more likely to be similar in terms of economic and social characteristics, therefore the results could be synthesized to inform interventions in HICs. 

The exclusion criteria were: (1) Studies not written in the English language. (2) Studies which examined the relationship between PA and risk factors for sight loss or eye disease e.g., the relationship between ocular perfusion pressure and PA. (3) Studies which compared PA between populations with sight loss and populations without sight loss and did not report the factors associated with PA within the population with sight loss. There were no further restrictions applied to the population of study i.e., studies were included regardless of the gender, ethnicity and reported comorbidities of the population group.

The data were independently extracted by one reviewer (R.K.L), and then discussed with a second reviewer (L.S). The data extracted included: age (years), the gender of participants, the sample size, the PA measurement tool, the vision measurement tool, the eye disease examined (if applicable), the statistical test used, the country in which the participants were recruited from, the confounders controlled for when examining correlates of PA, and the main findings.

## 3. Results

The database search yielded 2854 results; of these citations, 792 duplicates were removed. The grey literature was also searched, and no eligible studies were found. The primary reason no eligible studies were found in the grey literature search was that no studies reported the significance level of the variables which were associated with PA participation. A search of key terms related to physical activity in the British Journal of Visual Impairment, and The Journal of Visual Impairment yielded 112 and 71 articles, respectively. 

**Figure 1:**
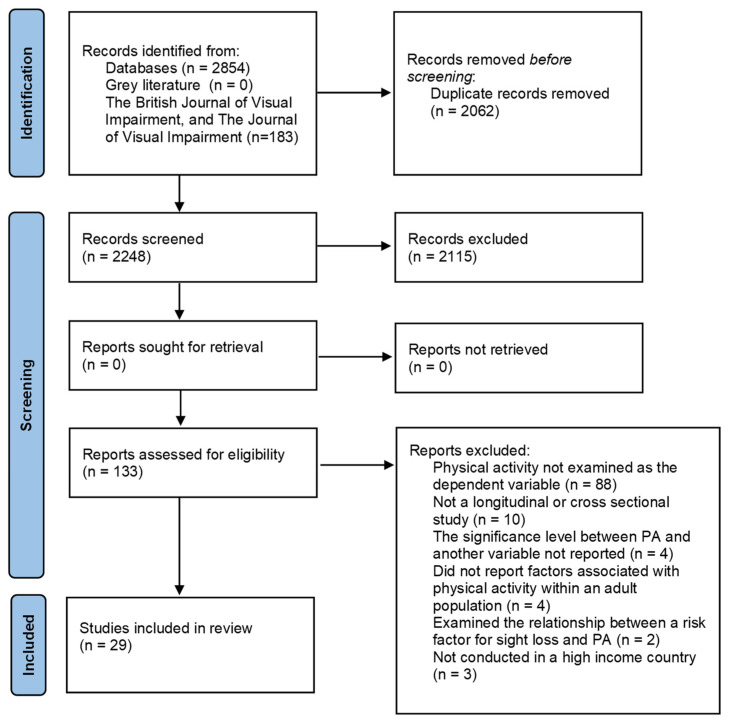
PRISMA flow diagram.

Following the abstract and title screening, and the subsequent full text screening, 29 studies were retained for the final review [7,18,19,20,21,22,23,24,25,26,27,28,29,30,31,32,33,34,35,36,37,38,39,40,41,42,43,44,45] (Figure 1). The data from eligible studies was extracted and reported in Table 1. 

The Critical Appraisal Skills Programme (CASP) checklist [46] was completed for each study and informed the final quality assessment; the results are presented in Table 2. Out of all eligible studies, 4/29 were considered to be of a high quality, 14/29 studies were of a medium quality, and 11/29 of the studies were considered to be of a low quality. Studies were more likely to be considered of a higher quality if they had minimised the risk of selection bias, had recruited a sample size which allowed for reliable conclusions to be made regarding the statistical significance of associations, and controlled for confounding factors, such as age and gender, in the analysis. Studies which relied on objective measures of PA and included a clinical assessment of vision parameters to categorise participants were also more likely to be considered of a higher quality than studies which used self-reported methods of PA and sight loss.

### 3.1. Non-Modifiable Correlates 

#### 3.1.1. Measures of Vision 

A range of different vision parameters and their association with MVPA were examined (Table 3), including self-reported VI classification (5/29), visual acuity in the better eye (3/29), visual field (5/29), contrast sensitivity (5/29) and colour vision (1/29). For studies which examined the association between self-reported VI classification and MVPA, there was evidence that being classified as blind was associated with lower levels of PA when compared to being classified as VI [21,31]. However, there was no evidence across studies of a dose–response interaction between the severity of sight loss, based on the international blind sports classification of VI (B1 vs. B2 vs. B3 vs. B4), and MVPA [24,27,42]. All of the studies which examined the relationship between MVPA and VI classification relied on self-reported MVPA measurement tools. In addition, it is possible that three of the studies may have selected the same participants for multiple studies, due to the similarities in the methods used in the recruitment of participants [21,24,25]. 

In contrast, among studies which used objective measures of sight loss and of MVPA, there was stronger evidence from one high-quality study, two medium-quality studies and one low-quality study that visual field loss in the better eye, or in both eyes, was significantly associated with lower levels of MVPA [37,38,44,45] However, unilateral visual field loss was not associated with MVPA [44]. Another study used a principal component analysis with varimax rotation to establish three independent factors; the first factor, which loaded on to superior visual field measures, and the second factor, which loaded on to inferior visual field measures, were also not associated with self-reported MVPA [19]. One study reported that the binocular visual field was associated with MVPA when MVPA was objectively measured, but not when MVPA was self-reported in the same group of participants [45].

The most frequently studied vision parameters that were associated with walking were visual acuity in the better eye (3/29), visual field (3/29) and contrast sensitivity (3/29) (Table 4). Worse measures of visual field in the better eye [38], bilateral visual field [44] and integrated visual field (IVF) sensitivity, which was defined as a summary of the average overall and inferior field sensitivities [39], with a lower IVF representing a worse visual field, were associated with lower levels of walking.

Studies which explored the association between visual acuity in the better eye and walking were all considered medium-quality studies and reported mixed results. One study, which used objective measures of walking, reported a significant negative relationship between visual acuity and daily steps taken [40]. In contrast, two studies which examined walking reported different outcomes which were dependent on the nature of the walking, or the group, examined. One study that reported walking was not associated with visual acuity in the better eye when the weekly time walked was examined, however, there was a negative association between visual acuity in the better eye and daily stairs taken [43]. Another study reported that the self-reported visual acuity status was a discriminating factor in walking distance for those over the age of 77, with a breaking point for those under and over 87.5 years [36].

The studies in the review reported mixed results for the association between PA and contrast sensitivity. Worse contrast sensitivity was found to be associated with lower levels of objectively measured MVPA in two studies, [40,45] and self-reported MVPA in one study [19]. However, contrast sensitivity was also found to have no association with objectively measured MVPA in two studies [37,38], and self-reported MVPA in one study [45]. For walking, there was no significant association found in any of the three studies between contrast sensitivity and objectively measured walking [38,40,45].

#### 3.1.2. Personal Correlates

In terms of the variables classified as non-modifiable personal correlates, twelve variables were tested for their association with MVPA (Table 5), and six variables were tested for their association with walking (Table 6). The most frequently reported correlation was between PA and gender. Overall, three studies reported that male participants self-reported engaging in significantly more MVPA than female participants [23,27,42], and five studies reported that there was no association between self-reported MVPA and gender [21,22,24,29,32]. One low-quality study examined the relationship between objectively measured MVPA and gender and reported no association between gender and MVPA [35]. For walking, two low-quality studies, one that relied on self-reported walking and the other that relied on objectively measured walking, both reported no association between walking and gender [35,42].

Older age was not found to be associated with self-reported MVPA in any of the four studies which examined MVPA [21,22,24,29]. One study reported that older age was associated with participants who self-reported less walking, however, the influence of age on walking was dependent on the sub-group examined. Among younger participants with poorer sight and who were more active, age was reported to have a greater impact on walking than sight loss [36]. No studies used objective measures of MVPA or walking to examine the association between PA and age.

Comorbidities, BMI, health related quality of life, depression, and level of independence were also included as non-modifiable personal correlates. These factors could have a bidirectional relationship with PA, which could also make them modifiable correlates. For example, improvements in depressive symptoms could help an individual feel more energised, and thus they are more likely to engage in PA.

### 3.2. Modifiable

#### 3.2.1. Personal Correlates

A range of psychosocial factors and their association with MVPA were examined, including self-efficacy [24,25] social support [21], self-regulation [21], the perceived barriers to PA [33], the theory of planned behaviour constructs (attitude towards PA, subjective norm, perceived behaviour control, intention to engage in PA) [22], use of a mobility aid [20], and levels of self-reported independence [23] (Table 7). Whilst only two studies, which were both conducted in similar cohorts, explored the relationship between MVPA and self-efficacy, the results from other studies provided evidence of factors which may also influence self-efficacy, defined as ‘the belief an individual has in their ability to perform a task and to obtain the desired results’. For example, a fear of falling was found to mediate the relationship between sight loss and PA in one study that was included in our review [37]. Perceived barriers to PA [33], and a lower self-reported level of independence [23] also had a negative and significant association with MVPA; it is plausible that these factors would influence an individual’s self-efficacy for PA. Social support was also reported to be positively associated with MVPA in one low-quality study [21], and peer/buddy support was also found to have a significant positive association with sports participation in a medium-quality study [30]. No studies explored psychosocial factors that were associated with walking.

#### 3.2.2. Environmental Correlates

When compared to the non-modifiable factors that were associated with MVPA, the modifiable environmental factors were less researched (Table 8). One low-quality study reported a negative correlation coefficient between the logit of the perceived barriers to PA and self-reported PA; included among the most severe barriers to PA which were cited by participants was the environmental barrier: ‘lack of transportation to get to places to exercise’ [33]. One medium-quality study found a positive association between access to services and non-walking PA, and a negative association between physical barriers to walking and non-walking PA [18].

Three studies examined environmental variables and their association with walking (Table 9). There was evidence from one medium-quality study, which used self-reported measures of walking, that the number of years lived at the same address was associated with increased walking, whilst feeling unsafe while walking around the neighbourhood was associated with less walking [36]. Another medium-quality study, which relied on self-reported walking measures, found no association between walking and neighbourhood aesthetics [18]. Only one low-quality study used objective measures of walking and found that there was no association between day of the week and walking [28].

Due to the variations between PA measurement tools and study design, it was not appropriate to conduct a meta-analysis for this review.

## 4. Discussion

Our review aimed to identify modifiable and non-modifiable correlates of PA among people with sight loss. Evidence from multiple studies reported that the VI category, worse visual acuity, bilateral visual field loss, worse contrast sensitivity, individuals of female gender, lower self-efficacy, and environmental barriers were associated with lower levels of PA among populations with sight loss.

Visual field and visual acuity are common measurements taken during a routine eye examination that is carried out by an optometrist or ophthalmologist. The measures of visual field and visual acuity are also used to classify people as blind or vision impaired. Our findings that worse visual acuity, bilateral visual field loss, and being classified as blind versus visually impaired could be associated with lower PA highlight that optometrists or ophthalmologists may be important for identifying populations at risk of low PA. Optometrists and ophthalmologists could therefore work with community groups and low vision services, to refer populations identified as being at risk of low PA to PA opportunities and support services. However, the vision parameters did not fully explain the variances in PA. Our review found additional non-modifiable and modifiable correlates of PA, which may have important implications for future PA interventions.

In terms of non-modifiable personal factors which correlate with PA, we found evidence that male participants were more likely to engage in higher levels of PA than female participants. These findings are in line with previous research in sighted populations. Globally, and particularly in HIC Western countries, men are, on average, more physically active than women [47]. However, one study in our review found that the relationship between gender and PA was mediated by social support and self-regulation [21]. Although these findings are limited to one study, which had a small sample size, the results highlight the importance of understanding the mechanisms which may lead to gender differences in PA. An intervention aimed at targeting low PA among women may not be effective if it does not target the mechanisms which result in women engaging in less PA than men.

Mixed results were found regarding the relationship between age and PA among people with sight loss. In sighted populations, research has consistently reported lower levels of MVPA that are associated with older age [48]. It is possible that because sight loss is associated with older age, the studies did not have a large enough sample of young adults with sight loss to be able to identify a negative association between older age and PA levels. Given that all age groups were identified as being at a possible risk of low PA, the findings highlight that multiple age groups could benefit from being targeted in PA interventions.

Importantly, our review also highlighted several areas where there is a lack of research. Firstly, there was limited research that explored the association between ethnicity and PA levels among people with sight loss. Among sighted populations, there is a large body of evidence that PA varies between ethnic groups within the UK [49,50] It is important to understand the PA differences between ethnic groups, to ensure that PA interventions do not compound the existing ethnic inequalities regarding the access to sight loss services [51]. In addition, there was a lack of research that explored the association between additional disabilities, as well as sight loss, and PA. Research suggests that additional disabilities are common among people with sight loss. For example, one in three people in the UK with a learning disability is estimated to be affected by a sight problem [52]. It is plausible that having multiple disabilities, including sight loss, may limit an individual’s opportunities and ability to engage in PA, thus increasing that individual’s risk of low PA. Therefore, it is important that interventions can be adapted to accommodate for people with additional disabilities, as well as sight loss. Further to this, there was a lack of research that explored the association between mobility measures and PA levels in populations with sight loss. Mobility measures may be modifiable by PA interventions, as research suggests PA programs could attenuate differences in gait and functional parameters between populations with sight loss and sighted populations [53]. Improvements in mobility among VI populations could also improve mental health outcomes, as previous research has found gait speed to be a significant predictor of depressive symptoms in VI populations [54]. Therefore, there is a need to understand the bidirectional relationship between PA and mobility measures within populations with sight loss, to determine how mobility measures should be targeted in an intervention to increase PA, and to determine target population groups (e.g., individuals with a slower gait speed).

The review also identified a range of psychosocial factors that are associated with low PA, including lower levels of social support, self-efficacy, intention to engage in PA, perceived barriers to PA, and lower levels of self-reported independence. There are a range of interventions which could be used to target these psychosocial factors; for example, group-based PA may encourage social support, whilst PA that is prescribed by a health professional could promote an intention to engage in PA. In addition, sight loss services may be able to support people in becoming more independent, and reduce the impact of barriers to PA, by supporting people in maximising their residual vision and improve daily functioning.

When compared to non-modifiable variables that were associated with PA, modifiable factors that were associated with the environment were less researched. Our review reported that an access to services, physical barriers, fears of safety, and perceived barriers, including ‘lack of transportation to get to places to exercise’, as well as familiarity with the neighbourhood, were associated with lower PA among people with sight loss. These could be considered barriers to PA which could be addressed by sight loss services; for example, orientation and mobility training could reduce individuals’ fears of safety in the neighbourhood. However, it is important that policies and planning also ensure that environments are designed to be accessible, and that the interventions to promote PA should consider environmental changes which can facilitate PA, as well as individual support to increase PA. Future research should also explore accessibility and mobility barriers which exist in low-income countries. It is plausible that the environmental barriers to PA that were identified in HIC countries in this review may be more pronounced in low-income countries, due to a lack of investment in public transport and infrastructure which make the streets more accessible, such as tactile paving, signal controlled pedestrian crossings, maintained pavements and detectable kerbs that separate traffic from pedestrians.

However, there were limitations to this review. Firstly, there were a limited number of studies which used objective measures of both sight loss and PA, which are considered more reliable and valid tools than self-reporting measurement tools. One study reported that in a group of patients with AMD, lower MVPA measured by objective tools was associated with worse visual acuity, visual field and contrast sensitivity, whereas when the same participants’ self-reported measures of PA were examined, the study did not report a significant negative correlation [45]. Although this study included a small sample size, the findings indicated the influence that a measurement tool can have on the outcome results. In addition, sight loss was defined and measured using a range of methods, and the studies used different criteria to define visual impairment and blindness, thus limiting the comparability of results. Future research that is conducted among populations with sight loss should adopt a standard definition of sight loss, to ensure the future comparability between studies. We suggest that sight loss can be defined as a self-reported ‘sight loss whilst wearing corrective devices’, however, a follow-up, standardised eye test should be used, if available, to describe the degree and type of sight loss of the population being studied. In the absence of eye testing equipment or expertise, then follow-up questions should be asked to understand the nature of the participants sight loss. Although some studies objectively measured vision and PA using validated tools, these studies were often limited by a smaller sample size than those which relied on self-reported measures. There were also limitations among studies as a result of the recruitment procedures. Studies which recruited participants via online channels that were distributed by a VI organisation risked selection bias, which resulted in younger and more active participants than the overall population of people living with sight loss. In addition, people with additional disabilities, in particular, cognitive impairments such as dementia and learning difficulties, may be excluded from studies if they are unable to understand the PA survey or provide informed consent. Therefore, the correlates of PA may not be representative of the barriers experienced by populations with the lowest levels of PA.

## 5. Conclusions

Overall, our findings have highlighted the complexities of the factors which are associated with PA behaviour among people with sight loss in HICs. Optometrists and ophthalmologists are well positioned to identify patients with sight loss who may be at risk of low PA, and collaborate with sight loss services (e.g., charities, community groups, and council services) to refer people to PA advice and support. In addition, people working in the delivery of sight loss services may be able to support people by addressing the barriers to PA, and promoting greater independence, which could facilitate PA. However, PA is complex and our review highlighted the need for PA interventions to meet the needs of a range of population groups with sight loss. We suggest that future research aims to understand how different sectors and services could identify people at risk of low PA, and work together to provide individualised support to promote PA.

### Implications for Practice

- Worse visual acuity and visual field may indicate that an individual is at risk of lower PA [29,36,37,38,40,43,44,45]. These measures are examined in routine eye tests, thus optometrists and ophthalmologists could identify people at risk of low PA, and play a key role in referring people to PA groups and opportunities.
- It is important to understand how factors including gender, age, ethnicity, and additional disabilities influence PA in the context in which interventions are being delivered. Interventions should work with communities to understand local needs, develop appropriate interventions, and target different sociodemographic groups, when appropriate [55,56].
- Interventions should consider the environmental factors, such as unsafe streets [41], and a limited access to services [18] which influence PA and make adjustments to minimise these barriers.
- Future studies, with larger, representative sample sizes, and objectively measured PA, are required, to explore the findings in studies which are currently limited to a small evidence base.

## Figures and Tables

**Table 1:** Characteristics of eligible studies.

Reference	Population Characteristics: Age (Years), Gender.	Population: (*n*)	Physical Activity Measurement Tool	Vision Measurement Tool	Specific Eye Disease Examined?	Statistical Test	Country	Confounders Controlled for	Main Findings
Barnett, A. et al. (2016) [18]	Mean age years (SD) age: 76 (6) Male: 34% Female: 66%	*n* = 527 (participant s with VI)	IPAQ-SF (Chinese version)	VI was determined using information from clinical health-problems checklists obtained from the Elderly Health Center (EHC). For participants recruited outside of the EHCs VI was self-reported.	Glaucoma Cataracts	The study examined if VI had a moderating effect on associations between perceived neighbourhood characteristics and physical activity outcomes.	Hong Kong	Socio demographics, type of recruitment centre, specific diagnosed chronic condition type, number of other medical conditions, and other significant perceived neighbourhood characteristics and environment by chronic condition interaction effects.	Land use mix–access to services was positively associated with non-walking PA. Physical barriers to walking were negatively associated with non-walking PA. Weekly frequency and minutes of within-neighbourhood walking for transport was not significantly associated with aesthetics in participants with VI.
Black, A. et al. (2011) [19]	Mean age (SD) years: 74.2 (5.9) Female: 47% Male: 53%	*n* = 74	Self-reported. Physical Activity Scale for the Elderly (PASE)	VA: Standard Bailey-Lovie high-contrast letter chart. VF: Humphrey Field Analyzer; model HFA-II 750; Carl Zeiss Meditec Inc., Dublin, CA, USA). Monocular 24-2 Swedish Interactive Threshold Algorithm (SITA)-Standard threshold tests.	Glaucoma patients.	Multivariate regression used to determine which vision parameters predicted variations in PASE scores. Principal components factors analysis with varimax transformation was used to create three vision factors which were assessed in multivariate analysis.	Australia	Age and gender	PASE scores were significantly associated with contrast sensitivity (r = 0.24) and all of the VF measures. The multivariate regression which examined vision factors and PA reported 10.2% of the variance in PASE scores was explained by vision factors, contrast factor was a significant predictor of PASE scores, whilst superior and inferior field factors were not.
Haegele, J. et al. (2016) [20]	Mean age years: 47.04 Female: 52.8% Male: 47.2%	*n* = 176	IPAQ-SF	Self-reported visual impairment classification	Any	Multiple regression analysis with total MET minutes per week as the dependable variable and sociodemographic variables as the predictors.	USA	Gender, ethnicity, VI type, onset, years of VI, K-12 education mobility aid and college education included in multiple regression.	Gender was the only significant predictor of MET minutes per week (β = 0.25, *p* < 0.05), with men reporting more MET minutes than women.
Haegele, J. et al. (2017) [21]	Mean age (SD) years: 46.88 (13.91) Female: 54.3% Male: 45.7%	*n* = 92	IPAQ-SF	Self-reported VI category. B1 (blind), B2 (travel vision), and B3 (legal blindness) = 28.	No	Hierarchical multiple regression analysis with forced entry	USA	Hierarchical multiple regression analysis controlled for vision status, sex and age.	The hierarchical regression found vision category (*p* < 0.001) and social support significantly predicted total METs (*p* = 0.037) but self-regulation (*p* = 0.094), sex (*p* = 0.069), and age did not.
Haegele, J. et al. (2017) [22]	Mean age (range) years: 45.3 (18–86) Female: 65.6% Male: 34.4%	*n* = 209	BAPS-VI scale	Participants self-reported as: B1, B2, B3 and B4 in accord with the United States Association of Blind Athletes classification system.	No	Hierarchical multiple regression analysis.	USA	Attitudes and beliefs variables, intention to engage in PA or sedentary behaviour, gender, age, and nature of VI (congenital versus acquired).	The model using theory of planned behaviour explained 7% of the variance in PA. Only intention to engage in PA resulted in a significant beta coefficient (β = 0.30).
Haegele, J. et al. (2017) [23]	Mean age (SD): 47.5 (12.4) Female: 62.5% Male: 37.5%	*n* = 80	Self-reported PA by the IPAQ-SF	Self-reported visual impairment. Participants had the option to select B1 (i.e., Blind), B2 (i.e., travel vision), or B3 (i.e., legal blindness).	No	Spearman rank correlation (to test ordinal variables), Pearson correlation (to test continuous variables).	USA	No confounders controlled for when conducting Spearman rank correlation testing.	Men reported higher levels of PA than women (*p* = 0.002). Level of independence and PA had a significant relationship (*p* = 0.003), and health related quality of life and PA (*p* = 0.010). Sedentary behaviour and PA were negatively correlated (*p* = 0.004).
Haegele, J. et al. (2018) [24]	Mean age (SD): 44.3 (15.3) Female: 65.2% Male: 34.8%	*n* = 147	IPAQ-SF	Self-Report visual impairment level. (B1, B2, B3)	No.	Linear multiple regression with forced entry for all independent variable to explore impact between variables and MET-min/week.	USA	Age, gender, VI classification and household income and self-efficacy included in the multiple regression analysis, MET-min/week was the dependent variable.	In multiple regression analysis self-efficacy was the only variable that reached significance as a positive predictor of MET-min/week (*p* = 0.001).
Haegele, J. et al. (2019) [25]	Mean age (SD) years: 44.77 (15.3) Male: 27.7% Female: 72.3%	*n* = 159	IPAQ-SF	Self-reported VI classification based on the United States Association of Blind Athletes system.	No.	Hypothesised structural model was tested.	USA	The structural model predicting quality of life, examined the direct and indirect paths predicting self-efficacy for exercise, physical health and psychological health and MVPA.	Self-efficacy for exercise positively predicted participants’ weekly MVPA (β = 0.26).
Haegele, J. et al. (2021) [26]	Mean age (SD) years: 44.8 (15.5) Male: 43.6% Female: 65.4%	*n* = 182	IPAQ-SF	Self-reported VI classification based on the United States Association of Blind Athletes system	No	Pearson product-moment correlation analysis. Hierarchical regression analyses	USA	No confounders controlled for in-person product moment correlation analysis. VI level, sex. Age, race, education level., body weight status, met MVPA guideline, met sedentary time guideline, and met sleep guidance were included in hierarchical regression analyses.	MVPA had a negative correlation with BMI (*p* < 0.05). The association between MVPA, and age, sedentary time, sleep and depression was not significant in correlation analysis. No significant association was found between meeting PA guidelines and depression in regression analysis.
Holbrook, E. et al. (2009) [27]	Age range: 18–60 Male: 60% Female: 40%	*n* = 25	Step Activity Monitor (SAM; Cyma, Seattle, WA; Model SW3).	Self-report VI severity based on the ICD classification	No	3 (mild, moderate, severe) × 2 (male, female) ANOVAs.	USA	The data was stratified by VI severity and by gender.	No interaction between the severity of VI and gender for four of the PA variables: average daily step counts, (*p* = 0.35) percentage of time at low activity, (*p* = 0.36) percentage of time at moderate activity (*p* = 0.93.)
Holbrook, K. et al. (2013) [28]	Mean age (SD) years: 45.9 (11.). Female: 61.3% Male: 38.7%	*n* = 31	Pedometer (Orbxy, Electronics Model 6310610, Concord, Canada)	Self-reported VI classification based on the International Statistical Classification for Disease schematic.	No	One-way repeated measures ANOVA Generalizability theory analysis	USA	PA stability was assessed across varying VI severity.	No difference in daily step activity across the days of the week in the whole sample (*p* = 0.633). 4 days monitoring was sufficient for a reliable measure of PA among people with mild-moderate VI and 9 days for people with severe VI.
Inoue, S. et al. (2018) [29]	Mean age (SD, range) years: 69.6 (14.5, 20–93) Male: 118 (54.9%)	*n* = 215	IPAQ-Japan	BCVA was assessed using clinical measures.	No	Univariate and multivariate ordinal logistic regression analysis to assess the association between physical activity and variables.	Japan	Sex, age, VFQ-25 score, BCVA in the better eye, and BCVA in the worse eye, systemic comorbidity, and BMI included in multivariate models.	Multivariate ordinal logistic regression analysis reported PA was significantly associated with VFQ-25 score (*p* < 0.001) and BCVA in the worse eye (*p* = 0.01) but not BCVA in better eye. Sex, age, systemic comorbidity, and BMI was not associated with PA in univariate or multivariate models.
Jaarsma, E. et al. (2014) [30]	Mean age (SD) years: 49.1 (17.9) Female: 52% Male: 48%	*n* = 648	Questionnaire.	Self-reported VI category based on ICD-10	No	Logistic regression (method enter) which included all variables related to sports participation (*p* ≤0.1).	The Netherlands	Education, use of white cane, use of computer software, having a guide dog, disability (experienced as a barrier), cost as a barrier, lack of peers/buddies as a barrier, and gender were entered as predictors of sports participation in the logistic regression.	The significant factors predicting sports participation were education, disability (experienced as a barrier), costs, lack of peer/buddies and use of computer software.
Jones, G. et al. (2010) [31]	Vision loss but not blindness group: Age 65–74: 43.5% ≥75: 56.6% Male: 38.5% Female: 61.5% Blindness: Age 65–74: 28.8% ≥75.5: 71.2% Male: 39.7% Female: 60.3%	Normal sight: *n* = 33, 497 Blind: *n* = 477 Vision loss but not blindness: *n* = 6721	Self-reported PA: Respondents were deemed physically inactive if they reported no regular weekly exercise.	Individuals who reported trouble seeing, even with glasses or contact lenses were classified as having vision loss. Participants were asked if they were blind or could not see at all. This was the case definition of blindness.	No	Logistic regression	USA	Age, sex, race/ethnicity, income, education, marital status, and correlated health behaviours.	There was a strong association between physical inactivity and severity of sight loss. 75.9% of adults with blindness did not exercise weekly (Adjusted odds ratio = 2.24), compared to 61.2% of adults with vision loss and not blindness (Adjusted odds ratio = 1.25).
Łabudzki, T. et al. (2013) [32]	Mean age (SD): 38 (±12.1) Female: 52.4% Male: 47.6%	*n* = 82	IPAQ-SF	Self-reported prior medical diagnosis of VI (significant, moderate or light impairment).	No.	Chi- square test for gender difference.	Poland	None controlled for when testing for differences in PA between genders.	No difference in PA in relation to gender (Chi square = 0.256, *p* = 0.88).
Lee M. et al. (2014) [33]	Age groups: n 20s: 20 30s: 16 40s: 23 50s: 52 >60s: 33 Female: 66.6% Male: 33.3%	*n* = 144 with VI/blind.	PASIPD	Self-report VA	No	Descriptive statistics used to screen the data. Pearson Product Moment Correlations (to examine relationship between PA participation and PA barriers).	USA	Confounders not controlled for when examining the relationship between PA and self-reported barriers to PA.	PA in the normal weight group was higher than in the overweight/obese group (t = 2.09, *p* < 0.04). A significant correlation (r = 0.41) was reported between PA levels and the logit of reported barriers to PA.
Loprinzi, S. et al. (2013) [34]	Dual sensory impairment sample: Mean age (range): 73.7 (58.7–88.7) Female: 59.5% Male: 40.5%	All participants: *n* = 1445 Mild/worse hearing loss: *n* = 817 VI: *n*= 35 Dual sensory impairment *n* = 29	Accelerometer (ActiGraph 7164; ActiGraph LLC)	Visual acuity (VA) assessed by an autorefractor. VA in the better eye worse than 20/40 after autorefraction or who self-reported not being able to see light with both eyes were classified as VI.	No	Negative binomial regression model	USA	Age, sex, race/ethnicity, education, body mass index, comorbidity index, cotinine level, C-reactive protein level, number of valid days of accelerometry, and accelerometer wear time.	Dual sensory impairment was associated with less PA compared to those with a single sensory impairment (Hearing loss × vision loss interaction: IRR, 0.45; 95% CI, 0.29–0.68, *p* = 0.001).
Marmeleira, J. et al. (2014) [35]	Mean age (SD) years: 47.4 (11.3) Male: 41 Female: 22	*n* = 63	Accelerometery(model GT1M; ActiGraph, Fort Walton Beach, FL)	People who were declared as legally blind by the Associatjao dos Cegos e Amblfopes de Portugal (The main Portuguese association for people with a VI).	Any	Independent sample t test or the nonparametric Mann-Whitney U test were used to compare PA between genders and congenital/acquired blindness groups. Chi-square test compared the proportion of participants who achieved the recommended levels of PA by gender. One-way ANOVA compared PA between BMI categories. Pearson’s correlation test relationship between physical activity and age.	Portugal	Not controlled for.	No significant gender difference in any of the PA variables. No differences across PA variables across BMI categories, age, age of blindness onset, or when comparing PA between people with congenital or acquired blindness.
McMullan, I. et al. (2020) [7]	Mean age years: 63.57 Female: 55% Male: 45%	*n* = 8255	IPAQ-SF	Self-Report. Participants were asked ‘Is your eyesight (using glasses or corrective contact lenses) excellent, very good, good, fair, or poor?’	No	Path analysis	Republic of Ireland	Age, marital status, sex, self-reported health, education, employment, depression, history of high blood pressure, eye disease, diabetes, and cardiovascular disease, and disability of activities of daily living.	Self-reported sight loss did not directly affect PA. However, PA had a cumulative effect on future PA, via its effect on vision over 6 years.
Montarzino, A. et al. (2007) [36]	Mean age (SD): 80.15 (8.2) Female: 67% Male: 33%	*n* = 66	Travel questionnaire.	Recruited from an eye clinic.	Any	Kruskal Wallis 1-way analysis of variance was carried out to identify significant differences in walking distance across all acuity ranges at *p* < 0.01. Kruskal Wallis ANOVA was run to test variables were significant discriminators of walking distance. The significant variables were then placed in a binary logistic regression to predict walking distance.	UK (Scotland)	Regression tree analysis included age, visual acuity and safety concerns as predictors of walking distance in the model and prioritised their importance.	The main restrictions on walking are the age of the participant, vision in the better and worse eyes, and feelings of safety, however these factors varied by age.
Nguyen, A et al. (2015) [37]	Controls: Age, median (IQR) 69.4 (65.2, 72.8) Female: 61.7% Glaucoma: Age, median (IQR) 70.4 (66.4, 74.5) Female: 53% AMD: Age, median (IQR) 75.8 years (71.0, 78.3) Female: 57.1%	Controls: *n* = 59 Glaucoma: *n* = 83 AMD: *n* = 58	Accelerometer (Actical; Respironics, Inc., Adover, MA, USA)	VF: Humphrey 24-2 VF testing (Carl Zeiss Meditec, Dublin, CA). VA: ETDRS chart	Glaucoma and AMD	Separate univariate negative binomial analyses were performed with MVPA as the dependent variable to identify covariates to be further explored in multivariate analyses.	USA	Covariates included in model 1 multivariate analysis were CS, Age, Sex, Race, Education, and Comorbidity as independent variables. In model 2 fear of falling was included as a possible mediator between CS and MVPA.	For participants with AMD, the association between CS and PA was no longer significant once fear of falling was added to the model (*p* = 0.53). For participants with glaucoma related VI, VF loss remained a statistically significant predictor of physical activity once fear of falling was added to the model (*p* < 0.01).
Ramulu, P. et al. (2012) [38]	No glaucoma: Age: 69.3 Female (%): 62.1 Glaucoma: Age: 70.3 Female: 54.2%	No glaucoma: *n* = 58 Glaucoma: *n* = 83	Omnidirectional accelerometer (Actical; Respironics, Inc., Andover, MA, USA).	VA: ETDRS chart. CS: Pelli-Robson chart (under binocular conditions) VF: Humphrey 24-2 VF testing (Carl Zeiss Meditec, Dublin, CA).	Glaucoma	Univariate analysis and Multivariate analysis negative binomial regression models.	USA	Variables included in multivariate analysis: Glaucoma (present), severe Glaucoma (present), visual field, age, race, gender, education, comorbidities, depressive symptoms, BMI, cognitive ability.	When the extent of VF loss, visual acuity, and contrast sensitivity were included in the same multivariate models, only VF loss remained predictive of either MVPA or steps (*p* < 0.01 for both).
Ramulu, P. et al. (2019) [39]	Age mean (SD): 70.7 (7.6) Female: 48% Male: 52%	*n* = 230	Accelerometer (Actical, Respironics Inc., Murrysville, PA, USA)	VF: Humphrey Field Analyzer 24-2 test (Carl Zeiss Meditec, Inc., Dublin, CA, USA) VA: ETDRS chart CS: MARS chart	Glaucoma	Univariate and multivariable negative binomial models.	USA	Age, race, gender, number of comorbid illnesses, and polypharmacy.	In multivariable models, worse integrated visual field sensitivity, older age, female gender, African-American race and polypharmacy was associated with less daily steps (*p* ≤ 0.05 for all) greater comorbid illness was not associated with less daily steps (*p* = 0.41).
Sengupta, N. et al. (2015) [40]	Control group: Age mean: 69.3 (5.3) Female: 62.7% AMD group: Age mean: 74.4 (5.2) Female: 57.9%	AMD patients: *n* = 57 Controls with normal vision: *n*=59	Accelerometer (Actical; Respironics, Inc., Andover, MA, USA)	VA: ETDRS chart CS: Pelli-Robson chart	AMD	Univariate and multivariate negative binomial regression models.	USA	Multivariate negative binomial regression models adjusted for age, gender, race, comorbidities, and education.	A significant dose-dependent relationship was observed between worse clinical measures of vision and daily MVPA and daily steps (*p* < 0.05).
Shakarchi, A. et al. (2019) [41]	Age mean (±SD): 70 (±6.8) Female: 53% Male: 47%	*n* = 151	Accelerometer, (Actical, Respironics, Inc., Murrysville, PA, USA)	VA: ETDRS chart CS: MARS chart VF: Humphrey Field Analyzer 24-2 test (Carl Zeiss Meditec, Inc., Dublin, CA, USA) AULCSF: Estimated from the quick CS function (qCSF) test (Adaptive Sensory Technology, San Diego, CA, USA) Colour vision: Hardy-Rand-Rittler (OttLite Technology, Tampa, FL, USA) Stereoacuity: Distance Randot Stereotest (Stereo Optical, Chicago, IL, USA). ViN: Pelli-Levi Dual Acuity Chart	Glaucoma	Likelihood ratio testing determined tested association between PA and vision parameters. Dominance analysis determined the relative importance of the various visual parameters.	USA	Age, sex, race, marital status, living arrangements, employment status, and education. Polypharmacy (defined as having five or more non-eyedrop prescription) and comorbidities index.	Vision parameters significantly predicted daily steps *p* = 0.01. The dominant predictor of differences in daily steps was AULCF.
Starkoff, B. et al. (2017) [42]	Mean age (SD) years: 36.1 (13.9) Men: 48.7% Women: 51.3%	*n* = 115	IPAQ-SF	VI was self-report classification based on the International Blind Sports Federation and US association of blind athletes guidelines (B1, B2, B3, B4).	No.	One-way ANOVAs to assess PA differences between gender, BMI and extent of VI. To differentiate between blind and VI participants a 2X4 ANOVA was used to assess differences in PA between gender and BMI.	USA	Data was stratified by different types of PA (walking, moderate PA, MVPA and vigorous PA)	Males engaged in more moderate PA than females (*p* = 0.008). Walk time was significantly greater in participants who were visually impaired compared to those who were blind (*p* = 0.021). Overweight participants engaged in more vigorous PA compared to normal-weight participants (*p* = 0.020).
Subhi, Y. et al. (2016) [43]	No AMD: Mean age (SD): 70.5 (7.5) Female: 63% Male: 37% Early AMD Mean age (SD): 77.1 (5.6) Female: 52% Male: 48% Late AMD: Mean age (SD): 75.9 (7.6) Female: 64% Male: 36%	No AMD: *n* = 68 Early AMD: *n* = 25 Late AMD: *n* = 103	Self-report questionnaire.	BCVA in each eye was measure using Early Treatment of the Diabetic Retinopathy study chart.	AMD	Chi-Square test or Fisher’s exact test when numbers were small.	Denmark	Participants were age matched with healthy participants with no AMD.	No difference in between participants at different stages of AMD. People with worse BVCA in best-seeing eye and worse-seeing eye were more likely to report: not engaging in PA weekly, to report engaging in vigorous PA <1 times a week compared to ≥1 a week and walking ≤ 10 stairs steps daily compared to 11–50 stairs steps and > 50 stairs steps.
van Landingham, S. et al. (2012) [44]	Age years: 40+ Female: 53%	Normal VF: *n* = 1321 Unilateral VF loss: *n* = 88 Bilateral VF loss: *n* = 59	Accelerometer (Actigraph, LLC, Ft. Walton Beach, FL)	VF Humphrey Matrix FDT 19-point suprathreshold screening test (N-30-5). VA post refraction: Nidek Autorefractor/Keratometer (model ARK-760A)	No.	Multivariable negative binomial models to assess relationship between VF and PA. Multivariate analysis was also used to test association between other vision and sociodemographic factors on PA.	USA	Covariates included in the multivariable models were age, sex, race/ethnicity, and education. Medical comorbidities included in the multivariable models were chronic obstructive pulmonary disease/asthma, arthritis, diabetes, congestive heart failure, and stroke.	In multivariable models bilateral VF loss but not unilateral VF loss was associated with fewer daily steps (*p* < 0.05) and less MVPA (*p* < 0.01). Post-refraction VA was associated with fewer 15% daily steps (*p* = 0.04) and 36% less MVPA (*p* = 0.04).
Zult, T., (2020) [45]	AMD subjects with vision loss: Age (SD): 76 (7) Male: 45.5% Female: 54.5% AMD subjects without vision loss: Age: 76(7) Male: 60% Female: 50% Controls with normal vision: Age: 70(4) Male: 45.5% Female: 54.5%	AMD subjects with vision loss: *n =* 11 AMD subjects without vision loss: *n* = 10, Control group with normal vision: *n* = 11	Accelerometer (Actigraph GT3X tri-axial), activity monitor log and interview using the World Health Organisation: Global Physical Activity Questionnaire.	VA: Bailey-Lovie logMAR chart CS: Pelli-Robson chart VF: Humphrey Field Analyzer (Carl Zeiss Meditec Inc., Dublin, CA, USA) SITA-Standard 30–2 threshold test	AMD	Pearson’s correlation coefficients were calculated to assess whether there is a relationship between the severity of vision loss and outcomes of the Actigraph and GPAQ.	UK (England)	Control group was age matched with AMD group.	There was a significant negative correlation between objectively measured MVPA and worse VA, VF and CS. However, when self-reported MVPA and step count was examined, the associations were weaker and in the opposite direction, compared to when PA levels were objectively measured.

BMI: body mass index, VI: visual impairment, PA: physical activity, IPAQ-SF: international physical activity questionnaire short form, PASE: physical activity scale for the elderly, MET: metabolic equivalent of task, BAPS-VT: Beliefs about Physical and Sedentary Behaviours-Visual Impairment, ICD: international classification of disease, VFQ-25: visual function questionnaire, VA: visual acuity, VF: visual field, CS: contrast sensitivity, BCVA: best corrected visual acuity, AMD: age related macular degeneration, MVPA: moderate to vigorous physical activity, PASIPD: Physical Activity Scale for Individuals with Physical Disabilities, GPAQ: global physical activity questionnaire, ETDRS: Early Treatment Diabetic Retinopathy Study chart (Available online: https://clinicaltrials.gov/ct2/show/NCT00000151, accessed on 5 November 2021), MARS chart: Mars Perceptrix, Chappaqua, NY chart.

**Table 2:** Quality assessment.

High Quality	Medium Quality	Low Quality
Loprinzi, S. et al. [34] Shakarchi, A. et al. [41] Ramulu, P. et al. (2019) [39] van Landingham, S. et al. (2012) [44]	Barnett, A. et al. [18] Black, A. et al. [19] Haegele, J. et al. (2017) [23] Haegele, J. et al. (2021) [26] Inoue, S. et al. [29] Jaarsma, E. et al. [30] Jones, G. et al. [31] Marmeleira, J. et al. (2014) [35] McMullan, I. et al. (2020) [7] Montarzino, A. et al. (2007) [36] Nguyen, A et al. (2015) [37] Ramulu, P. et al. (2012) [38] Sengupta, N. et al. (2015) [40] Subhi, Y. et al. (2016) [43]	Haegele, J. et al. (2016) [20] Haegele, J. et al. (2017) [21] Haegele, J. et al. (2017) [22] Haegele, J. et al. (2018) [24] Haegele, J. et al. (2019) [25] Holbrook, E. et al. (2009) [27] Holbrook, K. et al. (2013) [28] Łabudzki, T. et al. (2013) [32] Lee M. et al. (2014) [33] Starkoff, B. et al. (2017) [42] Zult, T., (2020) [45]

**Table 3:** Measures of vision and association with MVPA.

Measure of Vision	Positive Association		Negative Association		No Association	
Self-Reported	SR. PA	Obj.PA	SR. PA	Obj. PA	SR. PA	Obj. PA
Self-reported VI classification (blindness vs VI)			[21,31]		[42]	
Self report VI classification (B1, B2, B3,B4)					[20,24,42]	
Onset of VI (congenital vs after birth)					[20,22]	[35]
PA has an accumulative effect on PA over time via its effect on vision	[36]					
Years of VI					[20]	[35]
Self-rated vision					[36]	
**Objective Measures**	**Positive Association**		**Negative Association**		**No Association**	
	**SR. PA**	**Obj. PA**	**SR. PA**	**Obj. PA**	**SR. PA**	**Obj. PA**
Contrast sensitivity (worse)			[19]	[40,45]	[45]	[37,38]
Colour vision						[41]
Visual acuity without noise						[41]
Visual acuity (best seeing eye) (worse)				[40,45]	[29,43,45]	
VA (worse seeing eye) (worse)			[29]		[43]	
Visual field (worse)				[37,38,44,45]	[19,45]	[44]
Glaucoma (present)						[38]
Severe Glaucoma (present)				[38]		
Stage of AMD					[43]	
AMD present				[40]		
Significant cataracts/PCO (present)				[40]		

**Table 4:** Measures of vision and association with walking.

Measure of Vision	Positive Association		Negative Association		No Association	
Self-Reported	SR. PA	Obj. PA	SR. PA	Obj.PA	SR.PA	Obj.PA
Vision loss (self-reported blindness vs. self-reported VI)			[42] *			
Self-report VI classification			[42] **			
Onset of VI (Congenital vs. acquired blindness)						[35]
Years of VI/Age of onset						[35]
Severity of VI × Gender						[25]
**Objective Measures**	**Positive Association**		**Negative Association**		**No Association**	
	**SR. PA**	**Obj.PA**	**SR. PA**	**Obj. PA**	**SR. PA**	**Obj. PA**
Contrast sensitivity (worse)						[38,40,45]
Visual acuity (Best seeing eye) (worse)			[36] *** [43] (stairs taken daily)	[40]	[36,43], ***	
Visual acuity (Worse seeing eye)			[43] (stairs taken daily)		[43]	[44]
Visual field (worse)				[39] (Integrated visual field sensitivity) [38] (visual field loss in better eye) [45]		[45] (unilateral visual field loss was not associated with less steps) [44]
Vision parameters						[41]
Glaucoma (present)						[38]
Glaucoma (severe) (present)				[38]		
AMD (present)		[40]				
Stage of AMD					[43]	
Sig. Cataract/PCO						[40]

* Participants classified as B2 spent significantly more minutes of walking than participants classified as B1. ** Strakoff, B.E. (2017) reported a significant difference in walking between participants who self-reported VI categories B1 vs. B2 vs. B3 vs. B4. The mean min/day of participants classified as B1 was 46.8 min, com-pared to 95.8 min for participants in B2. However, there was no dose response identified as participants in the B3 category engaged in a mean of 62.6 min per day of walking. *** In regression analysis self-reported VA diagnosis, was a discriminating factor in walking for those over the age of 77 with a breaking point for those under and over 87.5 years.

**Table 5:** Personal non-modifiable variables and their association with MVPA.

Personal Characteristics	Positive Association		Negative Association		No Association	
	SR. PA	Obj. PA	SR. PA	Obj. PA	SR. PA	Obj. PA
Comorbidities					[29]	
Hearing loss (dual sensory impairment)				[34]		
BMI (Higher)			[26,33]		[24,29,42]	[35]
Use of a mobility aid					[20]	
Health related quality of life (higher)	[23]					
Depression					[26]	
Level of independence (Higher)	[23]					
Age (older)					[21,22,24,26,29]	
Gender (men)	[20,23,42]				[21,22,24,29,31]	[35]
Household income					[24]	
Ethnicity- (comparing Caucasian, African America, Asian, Hispanics and other ethnic groups)					[20]	
Education					[20]	

**Table 6:** Personal non-modifiable variables and their association with walking.

Personal	Positive Association		Negative Association		No Association	
	SR. PA	Obj. PA	SR. PA	Obj. PA	SR.PA	Obj.PA
Comorbidities						[39]
Polypharmacy (≥5 vs. <5 non-eye drop medication)				[39]		
BMI (Higher)					[42]	[35]
Gender (male)					[42]	[35]
Severity of VI × Gender						[25]
Age (older)			[36] **		[36] **	

** The influence of age on walking was dependent on the sub-group examined. Among younger participants, with worse vision and who were more active, age was reported to have a greater impact on walking than vision loss.

**Table 7:** Modifiable personal correlates of MVPA.

Personal Factors	Positive Association		Negative Association		No Association	
	SR. PA	Obj. PA	SR. PA	Obj. PA	SR. PA	Obj. PA
Self efficacy	[24,25]					
Self-regulation					[21]	
Social support	[21]					
Intention to engage in PA	[22]					
Attitudes/beliefs (theory of planned behaviour constructs)					[22]	
Sedentary behaviour (more time in SB)			[23]		[22,26]	
Level of independence (Higher)	[23]					
Use of mobility aid					[20]	
Fewer perceived PA barriers	[33]					
Sleep time (Higher)					[26]	

**Table 8:** Environmental factors associated with MVPA.

Environmental	Positive Association		Negative Association		No Association	
	SR. PA	Obj. PA	SR. PA	Obj. PA	SR. PA	Obj. PA
Fewer perceived PA barriers	[33]					
Land use mix- access to services (1 unit increase)	[18]					
Physical barriers to walking (1 unit increase)			[18]			

**Table 9:** Environmental Factors associated with walking.

Environmental	Positive Association		Negative Association		No Association	
	SR. PA	Obj. PA	SR.PA	Obj.PA	SR.PA	Obj.PA
Feeling of safety when walking in the neighbourhood (worse)			[36]			
Years lived at the same address (i.e., neighbourhood familiarity)	[36]					
Neighbourhood aesthetics					[18]	

## Data Availability

Not applicable.

## Appendix A

(“activities of daily living” OR “physical activity” OR “lifestyle activity” OR “inactive” OR “insufficientactivity” OR “mobility” OR “incidental activity” OR “walking” OR “active transport” OR “non exercise activity thermogenesis” OR “lipa” OR “lpa” OR “neat” OR “light exercise” OR “moderate exercise” OR “nepa” OR “sport” OR “exercise” OR “walking”) AND (Barrier* OR facilitator* OR modifier* OR motivator* OR influences OR uptake OR engagement OR correlate* OR encourage OR obstacle OR prevents OR participation OR predictor OR mediator OR moderator) AND (“Visually impaired” OR “Sight loss” OR “Sight impairment” OR “macular degeneration” OR AMD OR “Uncorrected refractive error” OR “Glaucoma” OR “Diabetes retinopathy” OR “Eye impairment*” OR “Visual impairment” OR “Vision disorder *” OR “Vision impaired” OR “low vision” OR “ocular pathology”).
